# Perceptual Video Coding Scheme Using Just Noticeable Distortion Model Based on Entropy Filter

**DOI:** 10.3390/e21111095

**Published:** 2019-11-08

**Authors:** Xin Cui, Zongju Peng, Gangyi Jiang, Fen Chen, Mei Yu

**Affiliations:** 1 Faculty of Information Science and Engineering, Ningbo University, No. 818, Ningbo 315211, China; cuixin2461@163.com (X.C.); jianggangyi@126.com (G.J.); yumei@nbu.edu.cn (M.Y.); 2 School of Electrical and Electronic Engineering, Chongqing University of Technology, Chongqing 400054, China; chenfen@cqut.edu.cn

**Keywords:** UHD image/video, just noticeable distortion, Gauss differential entropy filter, perceptual video coding, high efficiency video coding

## Abstract

Because perceptual video coding (PVC) can reduce bitrates with negligible visual quality loss in video compression, a PVC scheme based on just noticeable distortion (JND) model is proposed for ultra-high definition video. Firstly, the proposed JND model is designed, considering the spatial JND characteristics such as contrast sensitivity, luminance adaptation and saliency weight factor. Secondly, in order to perform precise JND suppression, the Gauss differential entropy (GDE) filter is designed to divide the image into smooth and complex texture region. Thirdly, through incorporating the proposed JND model into the encoding process, the transform coefficients are suppressed in harmonization with the transform/quantization process of high efficiency video coding (HEVC). In order to achieve the JND suppression effectively, a distortion compensation factor and distortion compensation control factor are incorporated to control the extent of distortion in the rate distortion optimization process. The experimental results show that the proposed PVC scheme can achieve a remarkable bitrate reduction of 32.98% for low delay (LD) configuration and 28.61% for random access (RA) configuration with a negligible subjective quality loss. Meanwhile, the proposed method only causes about average 12.94% and 22.45% encoding time increase under LD and RA configuration compared with an HEVC reference software, respectively.

## 1. Introduction

Ultra-high definition (UHD) video provides viewers with enhanced visual experience via a wider field of view (FOV) and more exquisite frames than high definition (HD) video [1]. UHD video is widely applied in various fields, education, amusement, sports, etc. [2,3]. Unfortunately, the large bandwidth and storage space are required for realization of these UHD video applications [4,5]. These problems limit the development and application of UHD video. Besides spatial and temporal redundancies, visual redundancy widely exists in UHD videos. Hence, the perceptual video coding (PVC) scheme can be utilized to further exploit the visual redundancy, and improve compression performance.

The key of PVC is to determine the distortion that users can just notice. Therefore, the study of just noticeable distortion (JND) and PVC become a hot topic and has attracted much interest. So far, various PVC schemes based on JND have been proposed and utilized for HD image/video compression [6,7,8,9,10,11,12,13,14,15,16,17,18,19,20,21,22,23,24,25,26]. The JND models can be categorized into two types: pixel and subband (e.g., discrete cosine transform (DCT), wavelet) domain approaches.

Specifically, the pixel-based JND profiles are established in the pixel domain, considering luminance masking (LM) and contrast masking (CM) effects [6,7,8,9]. Yang et al. proposed a pixel-based JND profile by considering the overlapping effect between LM and CM effects [6]. Chen et al. incorporated a foveated masking model into the JND profile and the JND values were weighted with visual eccentricity [7]. Liu et al. improved the work in [6], where the JND model was more precisely estimated by decomposing edge pixels from texture regions [8]. Wu et al. proposed a JND profile with free energy principle, where an autoregressive model was deduced to imitate the active prediction of the human visual system (HVS) with Bayesian inference [9]. Meanwhile, the disorderly concealment effect was estimated for the JND model. Several stereoscopic JND profiles have been established in the pixel domain [10,11,12]. Zhao et al. established a binocular JND profile by considering the asymmetric binocular vision property [10]. Zhang et al. proposed a JND profile for autostereoscopic display by considering the view position [11]. Wang et al. considered binocular spatial sensitivity in stereoscopic image quality assessment and utilized the [10] to measure the joint visibility of a pair distortions in the two views of stereoscopic image [12].

The pixel-based JND profiles cannot ensure conformance with perceptual characteristics of human eyes, because they do not individually take the sensitivity of HVS for each frequency component into consideration. In contrast, the subband-based JND profiles are modeled in subband domains and show better performance. In particular, the JND profiles in the DCT domain are more appropriate to image/video compression schemes [13,14,15,16,17,18,19,20]. Ahumada and Peterson proposed a JND based on DCT-domain quantization matrices which is known as the DCTune algorithm [13]. Watson applied the 8 × 8 JND-based DCT quantization matrix to a Joint Photographic Experts Group (JPEG) coder [14]. Wei and Ngan et al. proposed a CM-JND model based on 8 × 8 block. There were different CM-JND values for three image patch types of plain, edge, and texture [15]. Ma et al. extended [15] to work even at a 16 × 16 block-sized DCT kernel [16]. Luo et al. proposed a JND model to suppress the magnitudes of transform coefficients [17]. Bae et al. proposed a JND model in a DCT domain [18]. They proposed an elaborate JND model for variable block-sized transforms in a monochrome image [19]. They also incorporated foveated masking effects into a DCT-based total JND profile in [20].

Recently, many related works focus on the DCT-based JND profiles for high efficiency video coding (HEVC)-complaint PVC scheme [21,22,23,24,25,26]. Kim et al. proposed an HEVC-compliant PVC scheme based on JND models for variable block-sized transform kernels [21]. In addition, they proposed a compensation factor to tackle the distortions caused by transform coefficients suppression with JND profile. Bae et al. proposed an HEVC-based perceptually adaptive video coding method using a DCT-based local distortion detection probability (LDDP) model [22]. The proportion of suppression was modulated by the LDDP model. The models in [21,22] did not take the quantization parameter (QP) into account. Kim et al. proposed a JND model with the convolutional neural network. Moreover, the QP was considered in encoding process [23]. However, the distortion increased when the transform coefficients were suppressed in rate distortion optimization (RDO) process. Zhang et al. proposed a novel perception-based quantization to remove nonvisible information in high dynamic range (HDR) color pixels by exploiting luminance masking [24]. Through this method, the coding performance of HEVC was improved for HDR content. Jiang et al. proposed a saliency-based quantization control algorithm to reduce the bitrate of HEVC encoding processing [25]. Zeeshan et al. proposed a HEVC-compliant PVC scheme using entropy based visual saliency model to adjust the quantization parameter at the coding tree unit (CTU) level [26].

The conventional JND models are designed and utilized for PVC of videos with eight-bit depth. The existing JND models for videos with 10-bit depth are directly derived from the conventional JND models [6,7,8,9,10,11,12,13,14,15,16,17,18,19,20,21,22,23,24,25,26]. They are only a simple extension of the luminance range. In this work, we perform psychophysical experiments, and reveal that JND models with the simple extension do not conform well with perceptual distortion of images/videos with 10-bit depth. Hence, we proposed a JND model for the UHD images/videos with 10-bit depth based on psychophysical experiments. Following this, a perceptually HEVC-complaint PVC scheme based on the proposed JND model is proposed for improving the encoding efficiency of UHD videos with 10-bit depth. The main contributions of the work are as follows:

Because the conventional JND models are inappropriate for perceptual distortions of UHD videos with 10-bit depth, an LM effect is proposed. To consider the JND threshold values in the texture region, the Gauss differential entropy (GDE) filter is designed to divide the image into smooth and complex texture region. Because the visual focus is not fixed in a specific region when viewing the UHD videos, a saliency factor is incorporated into the proposed JND model. Moreover, the edge of the image is protected in the saliency region.

A perceptual friendly HEVC-complaint PVC scheme is proposed. Meanwhile, a distortion compensation factor (DCF) and a distortion compensation control factor (DCCF) are incorporated into the RDO process for improving the encoding efficiency of UHD videos with 10-bit depth.

The remainder of the paper is organized as follows. In Section 2, we introduce a new JND model that takes luminance masking effect of UHD videos with 10-bit depth, saliency factor, Gauss differential entropy filter into account. In Section 3, a HEVC-compliant PVC scheme is designed via the proposed JND model. Section 4 presents the subjective and objective experimental results for the proposed HEVC-complaint PVC schemes. Section 5 concludes our work.

## 2. The Proposed Transform-Domain Suppressed JND Model

**Figure 1:**
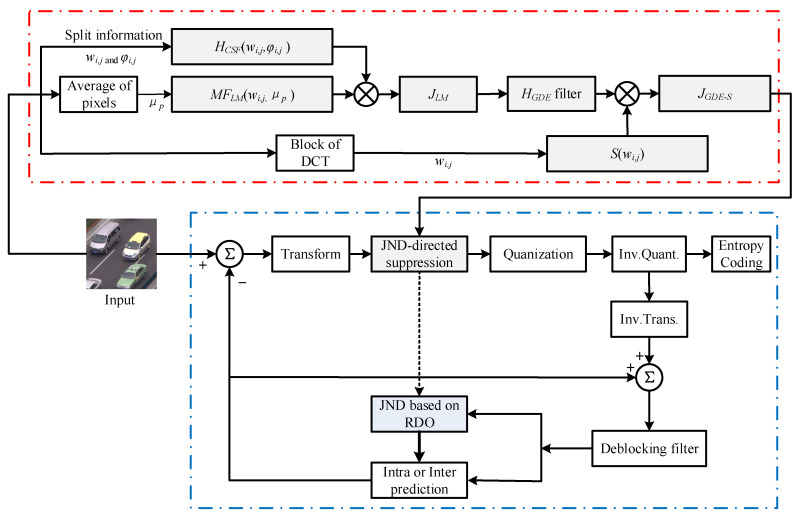
Overall framework of perceptual video coding with the JND suppression.

Usually, the SKIP mode is an efficient inter prediction technology, and it does not incorporate transforms in HEVC. Therefore, the proposed JND model based on a DCT domain is applied for the non-SKIP mode for various block sizes (4 × 4–32 × 32) of the coding unit (CU) and the transform unit (TU). Figure 1 shows the overall framework of the proposed HEVC-compliant PVC scheme. The symbols are described in Table 1. The PVC scheme includes two parts, the proposed JND model and original encoder. The block diagrams of the two parts are respectively surrounded by red and blue dotted boxes. The JND model is used to suppress the transform coefficients.

The “red dotted box” of Figure 1 shows the proposed JND model. To estimate the JND threshold values for transform coefficients suppression, the original TU blocks of input image are applied to calculate the *μ_p_* for the LM effect. Here, the LM effects are calculated as modulation factors. Then, LM effects are multiplied to a basic contrast sensitivity function (CSF). In order to control the proposed JND threshold values range in the smooth and complex region of image, the GDE filter is incorporated into *J_LM_* model. Finally, the *J_LM_* model after the filter multiply the saliency factor for the FOV of UHD.

To effectively apply the transform coefficient based on JND suppression, the DCF and DCCF are proposed to compensate the distortion introduced by *J_GDE-S_* model at different QPs. Following this, they are incorporated into the RDO process of HEVC in the “blue box” of Figure 1.

### 2.1. The Proposed JND Model by GDE Filter and the Saliency Factor

An appropriate transform-based JND model for UHD image/video with 10-bit depth is proposed in this section. Firstly, the basic framework of transform domain JND model is modeled based on the CSF and proposed 10-bit LM effect. Following this, the proposed JND threshold values are suppressed by the GDE filter. Besides, the saliency factor is incorporated into the proposed JND model.

#### 2.1.1. The Basic Framework of the JND Model

The proposed basic framework of JND model is constructed based on the *H_CSF_* and *MF_LM_*, which is expressed as:

(1) $$J_{LM}(w_{i,j},\varphi_{i,j},\mu_{p})=H_{CSF}(w_{i,j},\varphi_{i,j})\cdot MF_{LM}(\mu_{p})$$

where *w_i,j_* is cycles per degree (*cpd*) in spatial frequency for the (*i, j*)-th DCT coefficient, which is given by:

(2) $$w_{i,j}=(1/2M)\sqrt{{\left(i/\theta_{x}\right)}^{2}+{\left(j/\theta_{y}\right)}^{2}}$$

where *θ_x_* and *θ_y_* indicate the horizontal and vertical visual angles of a pixel. The *M* is the transform sizes (*M* = four to 32 for HEVC). The pixel aspect ratio is one for most of the display screens, and the *θ* is identical in the horizontal and vertical directions [15], which is given by:

(3) $$\theta_{x}=\theta_{y}=\theta =2\cdot \arctan(\frac{1}{R_{VH}\cdot H\cdot 2})$$

where *R_VH_* is the ratio of the view distance to the screen height, and *H* is the number of pixels in the screen height. From (2) and (3), it is found that the view distance or the pixel resolution of display screen increases, the spatial frequency also increases for each DCT component.

In (1), the directional angle *φ_i,j_* between *φ_i,0_* and *φ_0,j_* at the (*i, j*)-th DCT coefficient is taken into account for *H_CSF_* modeling. Usually, the HVS is more sensitive to the distortions along the horizontal and vertical directions (*i* or *j* = 0) than the diagonal (*i* = *j*) direction in spatial frequency, the character is called the oblique effect [27]. The *φ_i,j_* is given by:

(4) $$\varphi_{i,j}=\arcsin\left(\frac{2\cdot w_{i,0}\cdot w_{0,j}}{{w_{i,j}}^{2}}\right)$$

the *μ_p_* is the normalized pixel intensity of image block, which is defined as:

(5) $$\mu_{p}=(1/KM^{2})\sum_{y}^{M}\sum_{x}^{M}I(x,y)$$

where *I*(*x*, *y*) is the pixel intensity at (*x*, *y*).

(6) $$K=2^{bit}-1$$

where *K* is the maximum pixel intensity (255 in 8-bit image format and 1023 in 10-bit image format). 

The CSF quantifies the characteristics of human visual perception, which has different sensitivities at different spatial frequencies. In this paper, we adopt *H_CSF_* threshold in the transform domain [16], which is given by:

(7) $$H_{CSF}(w_{i,j},\varphi_{i,j})=\frac{1}{\phi_{i}\phi_{j}}\cdot \frac{\exp(cw_{i,j})/\left(a+bw_{i,j}\right)}{r+(1-r)\cdot \cos^{2}\varphi_{i,j}}$$

where *φ_i_* and *φ_j_* are the normalization factors for transform coefficients, which is given by:

(8) $$\phi_{k}=\left\{ \begin{array}{l} \sqrt{1/M},\;\;\;k=0\\ \sqrt{2/M},\;\;k>0 \end{array}\right.$$

where the model parameter values of *a* = 1.33, *b* = 0.11, and *c* = 0.18 are defined as [15] in (7).

#### 2.1.2. The Proposed 10-Bit Luminance Masking Effect

The LM effect is modeled as a human visual function by which JND threshold values change along with the average pixel intensity. The existing JND models only extends the luminance range to adapt the images/videos with 10-bit depth, but do not fully consider the visual difference with increasing the luminance range.

The proposed 10-bit LM effect is obtained with psychophysical experiments by increasing the distortion amplitudes of the DCT coefficients until subjects start to perceive the distortions. Bae et al. designed a subjective experiment to obtain a LM effect in DCT domain [18]. In this paper, the subjective experiment is followed as in [18], however there are two differences with Bae’s work. The first one is that Bae et al. added distortion into selected 15 DCT coefficients in a lower triangle frequency zone of 8 × 8 DCT and perceived the distortion in the DCT domain. However, we directly added the distortions into all DCT coefficients and perceived the distortions in the luminance domain. Our method can better reflect the distortions of the DCT domain coefficients. The other is that we test the subjective experiments in a wider luminance region (0 to 1023) than Bae’s (0 to 255). Therefore, in order to get a more perceptual friendly 10-bit LM effect, the following subjective experiments are carried out as

**Figure 2:**
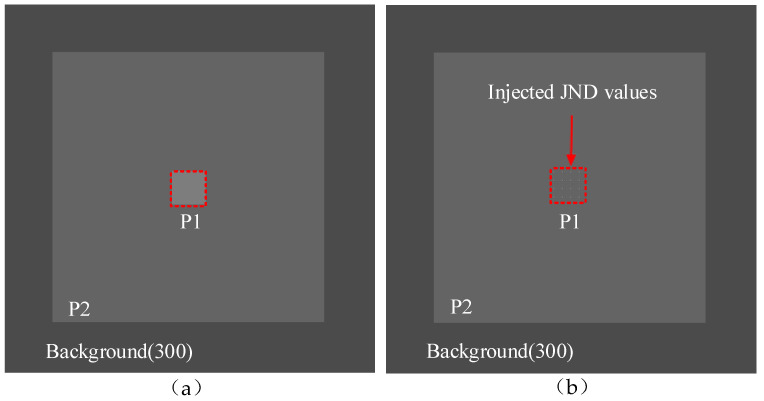
A test image exemplar, (**a**) a test image patch in a fovea region (**P1**), (**b**) the distortions injected into **P1**.

**Initialization**: Monitor shows test images resided in parafovea regions (**P2** in Figure 2). The experimental environments and conditions are listed in Table 2.

**Step 1:** A subject is noticed where the distortion will be injected by making the region dark (Figure 2a).

**Step 2:** The subject gradually increases the amplitude of distortion for the DCT coefficient until the viewer perceives the distortion in luminance domain, such as **P1** in Figure 2b. Each measured JND value is a value that 50% of subjects start to perceive as the corresponding distortion.

**Step 3:** Step 1 and Step 2 are iteratively conducted by going back to Step 1 with the different presentation, until all test presentations are finished.

**Figure 3:**
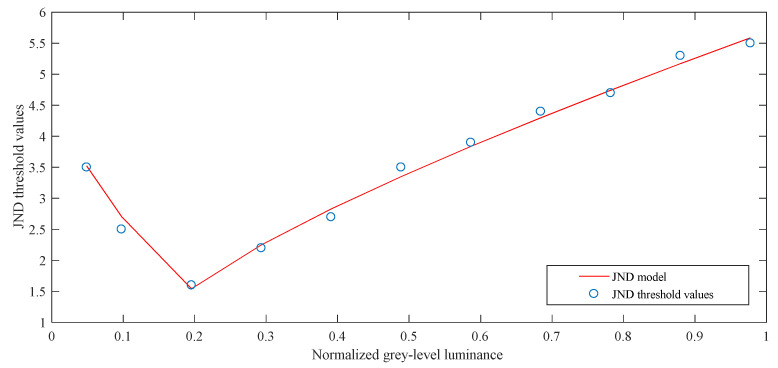
The subject experiment.

Figure 3 shows the experimental results of the subjective experiment. The circle represents the JND threshold values obtained from the subjective experiment. Normalized pixel intensity of the *N* × *N* TU blocks given by (5) are displayed in the *x*-axis and JND threshold values are in the *y*-axis. Zhang et al. exploit several parabolas to approximate the subjective data curve to build an LM effect model [28]. Therefore, we also use the method to fit subjective data, the proposed 10-bit *MF_LM_* model is expressed as:

(9) $$MF_{LM}(\mu_{p})=\left\{ \begin{array}{ll} A_{1}\cdot (1-{\left(\mu_{p}/0.2\right)}^{\alpha })+B, & \mu_{p}\leq 0.2\\ A_{2}\cdot {\left(\mu_{p}-0.2\right)}^{\beta }+B, & \mu_{p}>0.2 \end{array}\right.$$

where the parameters can be obtained by subjective experiments and data fitting, then *A1* = 4, *A2* = 5, *B* = 1.5, *α* = 0.5, and *β* = 0.8.

**Figure 4:**
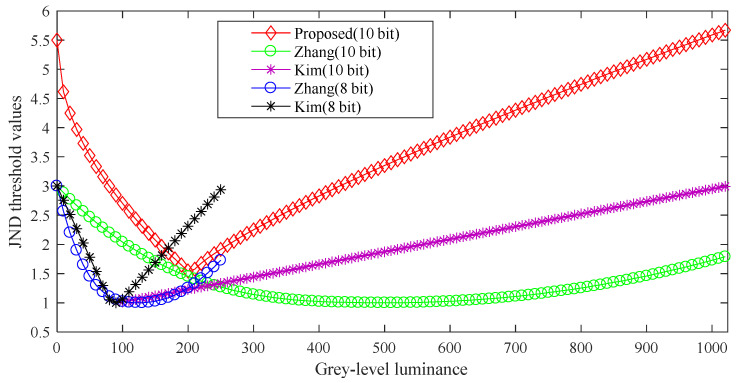
Comparison with the other luminance masking (LM) effect model in the grey luminance range.

Figure 4 shows that the proposed *MF_LM_* model compared with the proposed LM effect model by Zhang et al. and Kim et al. The luminance range of the Zhang’s and Kim’s is between 0 and 255, and the JND threshold values are between 1 and 3.2 in Figure 4. When the Zhang’s and Kim’s LM effect models are utilized to the 10-bit luminance range, the range of JND threshold values are consistent with 8-bit JND threshold values. Through subjective experiments, the proposed *MF_LM_* model does not only extend the luminance range, but also widens the JND threshold range than the existing LM effect models. As shown in Figure 4, the *MF_LM_* threshold values are between 1.5 and 5.7.

### 2.2. The Proposed 10-Bit JND Model Is Suppressed by the GDE Filter

With the dramatic improvement of UHD resolution, the image texture details are more fine and closer to the natural scene. To analyze the differences between pixels of UHD image/videos, the GDE is utilized in this paper. Meanwhile, the designed GDE filter is not only applied to divide the image into the smooth and complex texture region, but also it is utilized to control the extent of imperceptible noise in different regions of the image. Moreover, the texture of the image has not been considered in the proposed *J_LM_* model and the fixed quantization matrices are used, which limits the ability to perform precise JND suppression. The GDE is widely utilized in the information theory [29], and is generally defined as:

(10) $$H(x)=-\int_{-\infty }^{+\infty }p(x)\log_{2}p(x)dx$$

where *x* = *I*(*i, j*) expresses the (*i, j*)-th position pixel, *P*(*x*) is a probability density function for arbitrary pixels, which accord with the Gaussian distribution as follows:

(11) $$p(x)=\frac{1}{\sqrt{2\pi \sigma^{2}}}e^{-\frac{{\left(x-\mu \right)}^{2}}{2\sigma^{2}}},\;\;\;\;-\infty <x<+\infty$$

where *μ* is mean and *σ* is variance of the image block.

Therefore, (10) and (11) are utilized to deduced as:

(12) $$\begin{array}{ll} H(x) & =-\int_{-\infty }^{+\infty }p(x)\log_{2}p(x)dx\\ & =\frac{1}{2}\log_{2}(2\pi e\sigma^{2}) \end{array}$$

where the GDE is only related to the *σ*. Then, it is expressed as:

(13) $$H(\sigma )=\log_{2}\sigma +\frac{1}{2}\log_{2}(2\pi e)$$

**Figure 5:**
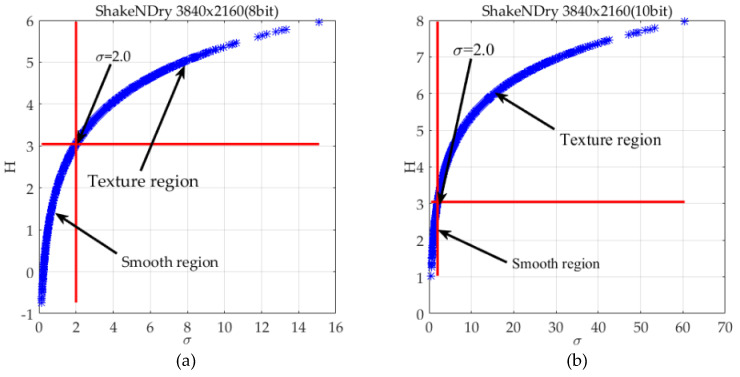
The GDE for one frame of ShakeNDry sequence; (**a**) 8-bit depth, (**b**) 10-bit depth

The *σ*-*H*(*σ*) curves of ShakeNDry video sequences with eight-bit and 10-bit depth are shown in Figure 5a,b, moreover they display an approximately logarithmic distribution. The small variance indicates that the texture of current image block is smooth and allows less imperceptible noise. On the contrary, the texture of the current image block is more complex and allows for more imperceptible noise. Thus, the *H*(*σ*) reflects the capacity of imperceptible noise in different regions.

The maximum curvature of the whole curve is taken as the “first-turning” point, which is used as the threshold to distinguish smooth regions from texture complex regions. Usually, let *ρ* be the curvature of the curve, and is expressed as:

(14) $$\rho =\frac{\left|H^{\prime \prime }(\sigma )\right|}{{\left(1+H^{\prime }{\left(\sigma \right)}^{2}\right)}^{\frac{2}{3}}}$$

where *H*′ and *H*″ are the first derivative and second derivative of *σ* respectively.

Finally, the “first-turning” point at (*σ_th_*, *ρ*(*σ_th_*)) which is the maximum curvature of the curve is obtained as follows:

(15) $$\left(\sigma_{th},\rho (\sigma_{th})\right)=Maximum\left(\rho \left(\sigma \right)\right)$$

where the *σ_th_* is a threshold to divide the image into complex texture and smooth region. In order to preserve more smooth regions, the “second-turning” point is defined as the threshold. The “second-turning” point is the maximum curvature of the curve from the “first-turning” point to the end, then (14) and (15) can be used to obtain as *σ_th_* ≈ 2.0 and *ρ* (*σ_th_*) ≈ 3.0, which are in the intersection of two red lines as shown in Figure 5a,b.

Table 3 lists statistics of image segmentation region. Specifically, the *σ* of image blocks is smaller than *σ_th_*, the current image blocks are in the smooth region, otherwise are in the complex texture or edge region. As shown in Table 3, the proportion of the smooth region of the video with 8-bit depth is larger than the texture region. However, for the video with 8-bit depth, the proportion of texture region of the UHD video is smaller than the texture region of HD video, because of UHD video containing more pixels in the same content image area. As shown in Table 3, the proportion of texture region is larger than smooth region for UHD image with 10-bit depth. Essentially, the UHD images can contain more texture details for the 10-bit grey luminance range.

Given the above analysis, the GDE filter is modeled as:

(16) $$H_{GDE}(x)=\left\{ \begin{array}{ll} H(x), & \sigma >\sigma_{th}\\ \sigma , & \sigma \leq \sigma_{th}\;\;\&\&\;\;H(x)\leq 0\\ H(x), & \sigma \leq \sigma_{th}\;\;\&\&\;\;H(x)>0 \end{array}\right.$$

**Figure 6:**
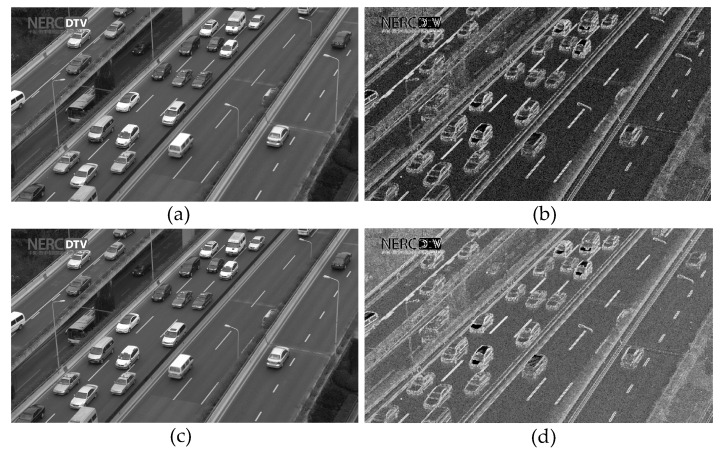
One frame of the TrafficFlow sequence is segmented by *H_GDE_* (**a**) original grey image with 8-bit depth, (**b**) segmentation image with eight-bit depth, (**c**) original grey image with 10-bit depth, (**d**) segmentation image with 10-bit depth.

As shown in Figure 6, one frame of the TrafficFlow video sequence is segmented by *H_GDE_* at 8-bit depth and 10-bit depth respectively. As shown in Figure 6b,d, the texture or edge is white region, and the smooth region is black region. Noticeably, there are more white regions in 10-bit segmentation image than in 8-bit segmentation image, since the image with 10-bit depth contains more texture details than image with 8-bit depth. Because the human eyes are not sensitive to the complex texture details, the complex texture details are essentially considered as noises for HVS. Thus, the image with 10-bit depth has a capacity for more perception distortions than the image with 8-bit depth. Meanwhile, the JND threshold values are mainly exploited in complex texture regions of the image.

The GDE is expressed as the capacity of noise in different regions of image. Therefore, the JND threshold values can be suppressed to a certain range by *H_GDE_* without causing perceptual distortion, so the *J′_LM_*(*μ_p_*) is obtained by *H_GDE_* filter and it is modeled as:

(17) $${J^{\prime }}_{LM}(\mu_{p})=\left\{ \begin{array}{ll} H(x), & \sigma >\sigma_{th}\;\;\;\&\&\;\;\;J_{LM}(\mu_{p})>H(x)\\ J_{LM}(\mu_{p}), & \sigma >\sigma_{th}\;\;\;\&\&\;\;\;J_{LM}(\mu_{p})\leq H(x)\\ \sigma , & \sigma \leq \sigma_{th}\;\;\&\&\;\;\;\;H(x)\leq 0\;\;\;\&\&\;\;J_{LM}(\mu_{p})>H(x)\\ H(x), & \sigma \leq \sigma_{th}\;\;\;\&\&\;\;\;H(x)>0\;\;\;\&\&\;\;J_{LM}(\mu_{p})>H(x)\\ J_{LM}(\mu_{p}), & \sigma \leq \sigma_{th}\;\;\&\&\;H(x)>0\;\;\;\;\&\&\;\;J_{LM}(\mu_{p})\leq H(x) \end{array}\right.$$

**Figure 7:**
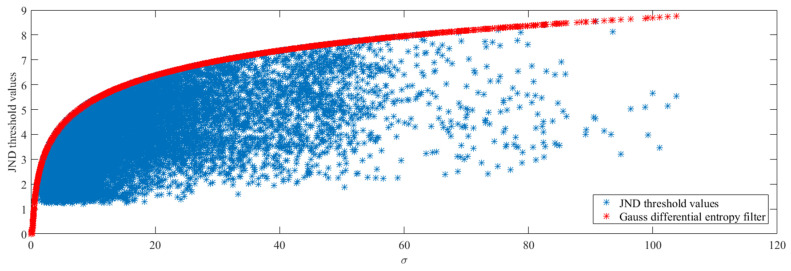
The JND threshold values distribution of TrafficFlow 3840 × 2160 video sequence with 10-bit depth.

As shown in Figure 7, the proposed *J_LM_* threshold values are “blue stars” and the *H_GDE_* threshold values are “red stars”. The proposed JND threshold values increase along with variances, but they all suppressed by *H_GDE_* with imperceptible distortions.

### 2.3. Saliency Factor

The human eyes do not view all UHD video contents. Furthermore, the visual focus will not be fixed in a specific region. Thus, the saliency region is utilized to simulate the change of visual focus when human eyes view the UHD image/video. Moreover, it better reflects the performance of the proposed JND model in a global image.

#### 2.3.1. Saliency Region Extraction Algorithm Based on DCT Domain

Hou et al. proposed a simple salient region extraction algorithm using the Fourier transform (FT) [30]. In order to maintain consistency with the HEVC coding structure and simplify the complexity of the algorithm, the DCT transform is used to replace the FT transform according to Hou’s method. Different from Hou’s method, the saliency region is modeled as:

(18) $$S(x,y)=g(x,y)\cdot DCT^{-1}{\left[\sin(R(u,v)+\theta (u,v))\right]}^{2}$$

where the *g*(*x*, *y*) is a gaussian filter, which is utilized to smooth the saliency map for better visual effects. The *DCT*^−1^(∙) is inverse discrete cosine transform. The residual spectrum of image *R*(*u*, *v*) is extract in spectral domain and is given by:

(19) $$R(u,v)=L(u,v)-h_{n}(u,v)\cdot L(u,v)$$

where *h_n_*(∙) represents the mean filter. The logarithmic spectrum of image on a log-log scale is defined as:

(20) L(u,v)=log(A(u,v))

*I*(*x, y*) represents one image, the amplitude spectrum of image *A*(*u, v*) is obtained by:

(21) A(u,v)=ℜ(DCT(I(x,y)))

and phase spectrum of image *θ*(*u, v*) is obtained by:

(22) θ(u,v)=ℑ(DCT(I(x,y)))

where *DCT*(∙) is denoted as a discrete cosine transform.

**Figure 8:**
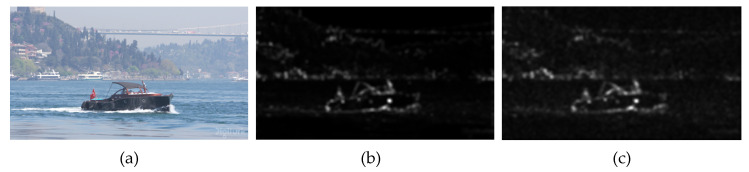
Comparison of the extracted saliency region with Hou’s and the proposed method; (**a**) original image, (**b**) based on FT, (**c**) based on DCT.

Figure 8b is the extracted saliency region based on Hou’s method and Figure 8c is the saliency region based on the proposed saliency extraction algorithm. As shown in Figure 8b,c, the saliency region of the proposed algorithm is consistent with Hou’s.

#### 2.3.2. The Proposed JND Model Based on the Saliency Weight Factor

Through using the proposed saliency region extraction algorithm, we continue to obtain the JND model based on saliency weight factor. Firstly, the object map is expressed as:

(23) $$\mu_{s}=\sum_{N}\sum_{N}S(x,y)/(N\cdot N)$$

where *μ* is *N* × *N* the normalized intensity of saliency.

(24) $$K_{th}=\beta \cdot \mu_{s}$$

The selection of *K_th_* is a trade-off problem between false alarm and the neglect of objects. According to [30] and subject experiments, we set *β* = 1.5 empirically. Thus, the object map *O*(*x, y*) in a saliency is defined as:

(25) $$O(x,y)=\left\{ \begin{array}{ll} 1, & if\;\;S(x,y)\geq K_{th}\\ 0, & otherwise \end{array}\right.$$

According to the characteristics of human subjective perception, small JND thresholds are utilized to saliency regions, while the large JND thresholds are utilized to non-saliency regions. Therefore, the saliency regions are inversely proportional to saliency weight factor *γ* and the *γ* is given by:

(26) $$\gamma =A-\alpha \cdot \mu_{0}$$

where the parameter *α* is measure factor. *μ*_0_ ∈ [0, 1] is normalized intensity of binary saliency image block with a size of *N* × *N* and expressed as:

(27) $$\mu_{o}=\sum_{N}\sum_{N}O(x,y)/(N\times N)$$

According to (17), the JND threshold values of the current image block are not larger than the *H_GDE_*. Thus, the conditional relation is obtained as:

(28) $$0<\gamma \cdot {J^{\prime }}_{LM}\leq {J^{\prime }}_{LM}\leq H_{GDE}(x)$$

where *J′_LM_* is suppressed by *H_GDE_*, then the range of values *γ* can be deduced as:

(29) 0<γ≤1

According to (28) and (29), the range of parameter α can be obtained, that is *α* ∈ [0, 1], then the parameter *A* can be deduced as:

(30) A=1

in order to obtain a large control range, the parameter *α* = 0.9.

Therefore, the JND model based on saliency weight factor is given by:

(31) $$J_{GDE-S}=\gamma \cdot {J^{\prime }}_{LM}$$

**Figure 9:**
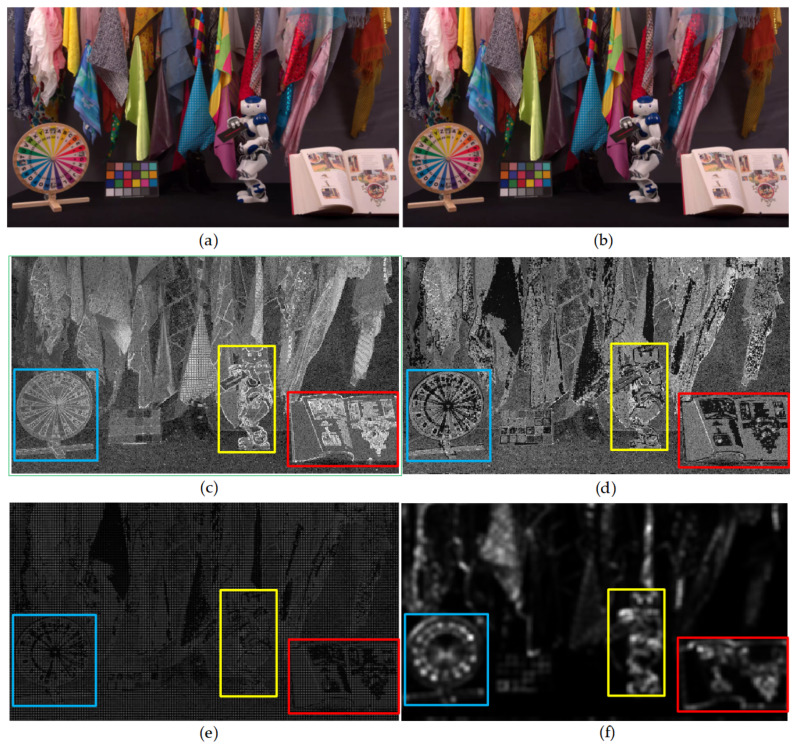
The fourth frame of the CatRobot1 (3840 × 2160) video sequence is contaminated by the proposed *J_GDE-S_* model. (**a**) Original image, (**b**) contaminated image, (**c**) *J′_LM_* image (without saliency weight factor), (**d**) *J_GDE-S_* image (with saliency weight factor), (**e**) difference image of (**a**) and (**b**), (**f**) saliency image.

As Figure 9b shows, one frame of CatRobot1 UHD video sequence is contaminated by the proposed JND values. Compared with Figure 9a, there are no obvious subjective visual distortion loss in Figure 9b. There are three colored (red, yellow and blue) boxes in Figure 9 to show the visual differences clearly. Figure 9c shows the distribution of *J′_LM_* threshold values, where the white regions represent that the JND values are more lager than the JND values in black or grey regions. Because the *H_GDE_* divides the image into complex texture regions and smooth regions, JND threshold values are mainly embedded in texture regions or edge regions of the image. Figure 9d shows the distribution of proposed *J_GDE-S_* threshold values. There are some saliency weight factors are incorporated into the *J′_LM_* model. Figure 9f shows the saliency image of Figure 9a, and as shown in Figure 9d, the edge region of saliency object is not embedded or embedded with small JND threshold values. Therefore, the edge regions of saliency object are protected to avoid the distortion of the edge region which could often cause significantly visual perception. Figure 9e shows that the difference region is the same as Figure 9b.

The temporal masking is not considered and incorporated into our proposed JND model through weighing encoding time and efficiency. Because the reuse of CU or TU block motion vectors are obtained in the HEVC recursively coding process, they are uncertain true motion vectors. Meanwhile, the precise temporal JND values are hard to calculate with these motion vectors, which may cause to bring additional visible distortions. Moreover, the calculation of the temporal masking model is computationally heavy because of the HEVC recursively coding process.

## 3. A JND-Based HEVC-Complaint PVC Scheme Using Distortion Compensation

### 3.1. Overall Architecture of the Proposed PVC Scheme

**Figure 10:**
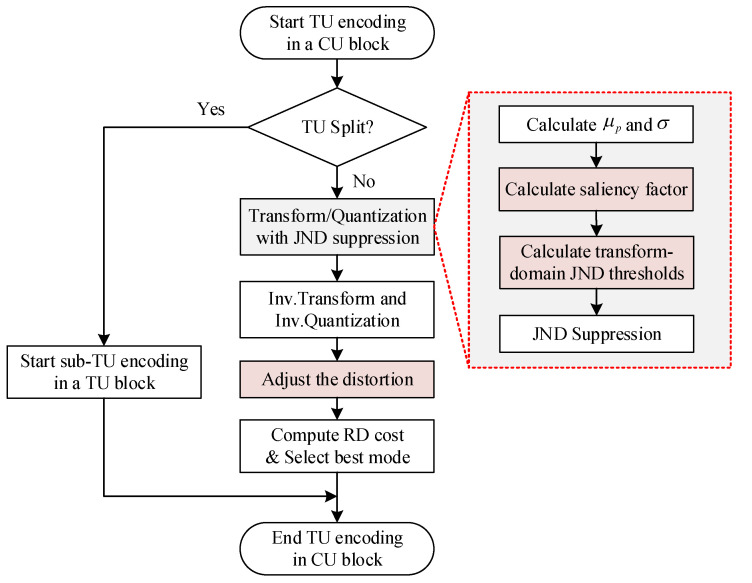
Flowchart of the proposed perceptual visual coding (PVC) encoder.

The proposed *J_GDE-S_* model is incorporated into an HEVC Test model (HM 16.9) reference software [31], and the JND-based perceptually HEVC-complaint video coding is proposed. Figure 10 shows the whole flowchart of proposed PVC encoder. Firstly, a CU block is split into some TU blocks according to the rate distortion (RD) cost. If the parent TU block is split into four sub-TU blocks further, then each sub-TU block is decided to continue splitting by RD cost of the parent TU and its children TUs respectively, else the transform coding process is carried out directly. The transform coding process is mainly divided into DCT transform and quantization. As shown in the “red dot box” of Figure 10, the average pixel value and the variance of the current *N* × *N* TU block is calculated, then the saliency factor is obtained in DCT domain, finally the proposed *J_GDE-S_* threshold is modeled.

**Figure 11:**
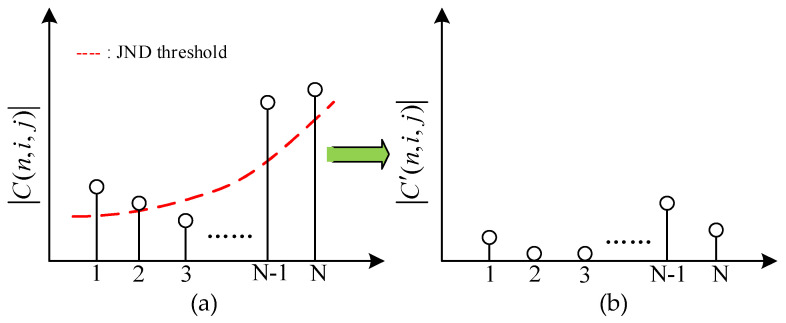
JND-directed suppression example for 1-D transform coefficients. (**a**) Before suppression, (**b**) after suppression.

Figure 11 shows the JND suppression example for 1-D transform coefficients. It is the distribution before JND suppression in Figure 11a, where the red dot line is represented the JND threshold. As (32) shows, if the amplitudes of transform coefficients |*C*(*n*, *i*, *j*)| < *J_GDE-S_*, then the after suppression transform coefficients |*C*′(*n*, *i*, *j*)| is set to zero. Otherwise, the |*C*′(*n*, *i*, *j*)| is equal to |*C*(*n*, *i*, *j*)| − *J_GDE-S_*. Through the above decision, the transform coefficients of complex texture regions or unperceived regions by the HVS are set to zero, and the transform coefficients of smooth regions or perceived regions are decreased by JND suppression. Since the zero or smaller transform coefficients are discarded in the quantization processing, the encoding bitrates are saved, and the coding compression efficiency is further to improved.

(32) $$\left|C^{\prime }(n,i,j)\right|=\left\{ \begin{array}{ll} 0, & \left|C(n,i,j)\right|-J_{GDE-S}\leq 0\\ \left|C(n,i,j)\right|-J_{GDE-S}, & otherwise \end{array}\right.$$

**Figure 12:**
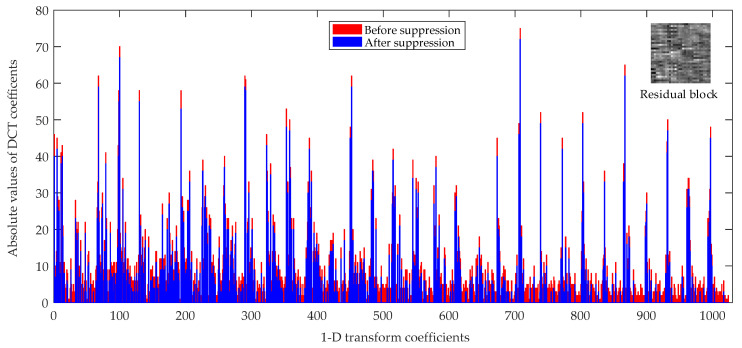
A 32 × 32 residual transform unit (TU) coding block of the BasketballPass video sequence under the random access (RA) main10 configuration at quantization parameter (QP) = 22.

As Figure 12 shows, the “red bars” are the absolute values of 1-D transform coefficients before suppression and the “blue bars” are the absolute values of 1-D transform coefficients after suppression with proposed JND threshold. As Figure 12 shows, we can draw the following two conclusions. First, the amplitudes of “blue bars” are all lower than the “red bars” because of the suppression with proposed JND threshold values. Second, the distributions of the “blue bar” are sparser than “red bars”, because the lower absolute values of coefficients than the JND threshold values are suppressed to zero. This explains that parts of the transform coefficients are suppressed by proposed JND threshold values.

### 3.2. The Proposed JND-Based HEVC-Complaint PVC Scheme using Distortion Compensation

Because the residuals of CU blocks are utilized in HEVC recursively coding process, the subsequent coding performance is influenced on the reconstructed image quality. For the transform coefficient based on the JND suppression method, a certain distortion of the reconstructed image will be produced, and then the coding errors are accumulated recursively in the encoding process. Therefore, how to control the distortion caused by JND suppression is the key to determining the final coding performance. Obviously, the distortions directly affect the RD cost of encoding process, and RD cost is the basis for judging the selection of encoding mode and CU partition. In (33), the RD cost function *J_RDO_* is composed of distortion *D*, bitrate *R* and Lagrange factor *λ*, which has a relationship with quantization step.

(33) $$J_{RDO}=D+\lambda \cdot R$$

Kim et al. control the distortion caused by JND suppression through incorporating the distortion compensation factor into RDO of the encoding process [21]. However, Kim’s method needs to calculate the dequantization DCT coefficients without JND suppression, which increases computational complexity. The proposed distortion compensation factor only needs to calculate the absolute value of the difference between the DCT coefficients before and after suppression. Moreover, the distortion compensation control factor (DCCF) under different QPs are introduced to control the extent of distortion compensation more efficiently.

(34) $${J^{\prime }}_{RDO}=1/\psi_{q}\cdot \varepsilon \cdot D+\lambda \cdot R$$

Equation (34) is the proposed rate-distortion (RD) cost function *J′_RDO_*, where *ε* is the distortion compensation factor (DCF) and *ψ***_q_** is DCCF.

The DCF is calculated by (35), where the *J_GDE-S_*(*n*, *i*, *j*) represents the (*i, j*)-th JND threshold values of the *n*-th block.

(35) $$\varepsilon =\sum_{N}\sum_{N}J_{GSE-S}(n,i,j)/\sum_{N}\sum_{N}\Delta C^{\prime \prime }(n,i,j)$$

The ∆*C*″(*n*, *i*, *j*) is the absolute value of the difference between transform coefficient before suppression and transform coefficient after suppression and is defined as (36). The DCF is large when ∆*C*″(*n*, *i*, *j*) is small, which means that the distortion caused by JND suppression is small at this time. Therefore, a large distortion *D* and small DCCF are allowed in the RD cost function. Otherwise the distortion caused by JND suppression is large, then the distortion *D* in the RD cost function needs to be reduced.

(36) $$\Delta C^{\prime \prime }(n,i,j)=\left|C^{\prime }(n,i,j)-C(n,i,j)\right|$$

**Figure 13:**
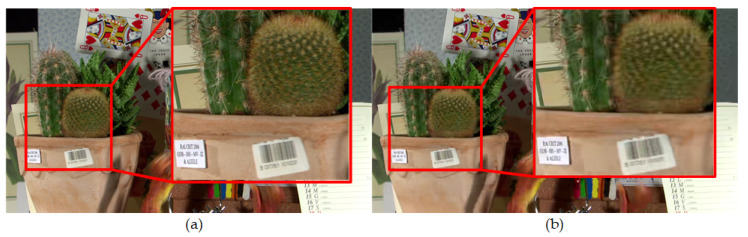
The 34th encoded frame of the Cactus video sequence with QP = 19 and QP = 40 under the RA mian10 configuration of HM 16.9. (**a**) The subjective and objective quality (PSNR) of the encoded frame is 40.10 dB with QP = 19; (**b**) the PSNR of encoded frame is 31.30dB with QP = 40.

Usually, the coding distortion of the video is smaller when using a small QP than the use of a large QP. As shown in Figure 13a,b, the subjective and objective (PSNR) quality of encoded frame when using QP = 19 is higher than that of encoded frame when using QP = 40. The encoding process causes high coding distortion because of using a large QP, and there is no chance to further suppress perceptual redundancy. Therefore, the large DCF is adopted to the RDO process. Otherwise, the small DCF is adopted to the RDO process and more bitrates are saved. Therefore, a DCCF is applied to control the extent of DCF under different QPs. When the QP value is greater than 40, because the objective and subjective quality of encoded video decreases significantly, the small DCCF is utilized. In order to get the relationship between DCCF and QPs, the designed decision is shown in (37), where q is a vector of QPs and **q** = [22 27 32 37]. Therefore, *ψ***_q_** represents the difference of PSNR between q ≠ 37 and q = 37 or the difference of PSNR between q = 37 and q = 40.

(37) $$\psi_{q}=\left\{ \begin{array}{ll} PSNR_{q}-PSNR_{37}, & q\neq 37\\ PSNR_{q}-PSNR_{40}, & q=37 \end{array}\right.$$

**Figure 14:**
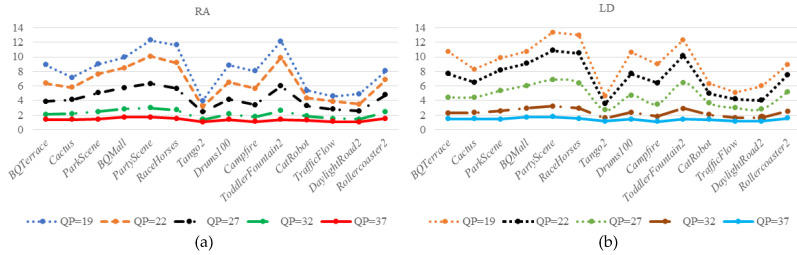
*ψ***_q_** with different QPs; (**a**) based on RA configuration, (**b**) based LD configuration.

In Figure 14, the statistics are come from different scenarios of HD and UHD sequences at different QPs under the random access (RA) and low delay (LD) configurations. As shown in Figure 14, we can obtain the values of *ψ***_q_** (in ordinate) are large at small QPs and the curves are smooth at high QPs. Essentially, the objective and subjective quality of encoded video are high at the small QP and more perceptual distortions are acceptable. Thus, the DCF is reduced and DCCF should be increased appropriately. On the contrary, in order to ensure the subjective and objective quality of the encoded video, and allow for small perceptual distortion, the DCF should be increased while the DCCF should be reduced.

**Figure 15:**
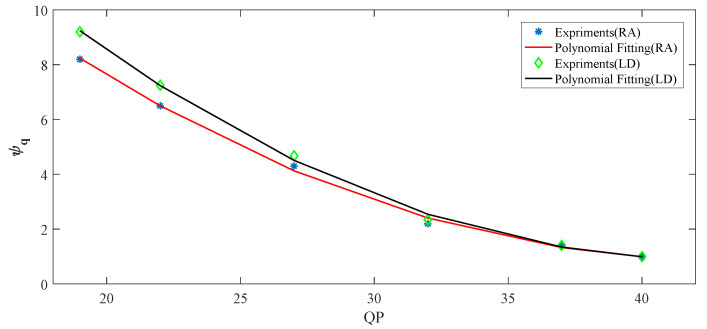
The polynomial fitting curves based on RA and LD configuration.

From the above statistical results, average values of *ψ***_q_** are set to DCCF under different QPs. Figure 15 shows the polynomial fitting curves based on the statistics in Figure 14. The *ψ***_q_** decrease with increasing QPs, and *ψ*_**q**_ is larger in LD configuration than in RA configuration, because the encoding usually operates with smaller prediction errors under the RA configuration than that under the LD configuration for different QPs. The *ψ***_q_** is given by:

(38) $$\psi_{q}=\left\{ \begin{array}{ll} 0.013\cdot QP^{2}-1.109\cdot QP+24.641, & for\;\;\;RA\;\\ 0.015\cdot QP^{2}-1.294\cdot QP+28.310, & for\;\;\;LD\; \end{array}\right.$$

where (38) shows the fact that the high *ψ***_q_** is utilized at small QPs. On the contrary, the small *ψ***_q_** is utilized at high QPs. It is consistent with the results shown in Figure 14.

## 4. Experimental Results

### 4.1. Verification of the Proposed J_GDE-S_ Model

**Figure 16:**
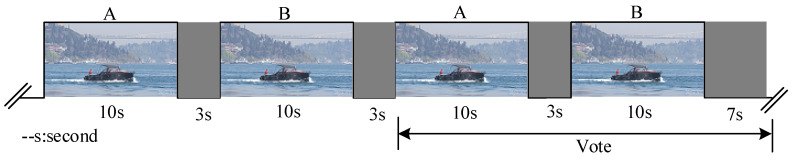
The double-stimulus continuous quality-scale (DSCQS) method for the movie video sequence.

The HD and UHD videos are mainly provided by [32] and Joint Video Exploration Team (JVET). For a wide FOV of 4K UHD video/image, it is not appropriate to apply the adjectival categorical judgment (ACJ) method, which shows the reference images (left) and test images (right) at the same time. To verify the effectiveness of the proposed *J_GDE-S_* model for 4K UHD video/image, subjective viewing tests are conducted based on double-stimulus continuous quality-scale (DSCQS) where A (a reference sequence) and B (a sequence to be compared) are pseudo-randomly ordered for each presentation [33,34]. For the subjective of still picture, the sequence A consists of one frame from 4K UHD video sequence. Then sequence A is contaminated by (40) to form sequence B as shown in Figure 16. However, a 3–4 s sequence and five repetitions (voting during the last two) may be appropriate for still picture [33,34]. The display condition is the same as the one in Table 2. The viewing distance is set to 2.1 m which is appropriate for a 55-inch 4K UHD display. A total of 15 subjects have participated in the subjective quality assessment experiments where all of them have normal vision power.

According to [33,34], the both A and B are evaluated with subjective voting scores ranged from 0 to 100 for the worst and the best visual qualities, respectively. The differential mean opinion score (DMOS) value is defined as:

(39) $$DMOS=MOS_{PVC}-MOS_{ORI}$$

where *MOS_PVC_* and *MOS_ORI_* are the measured mean opinion score (MOS) values from the image contaminated by the JND and the original image of sequence.

According to (40), the *C*(*n*, *i*, *j*) represent the (*i, j*)-th DCT coefficient of the *n*-th block, the *C*′(*n*, *i*, *j*) is the (*i, j*)-th DCT coefficient of the n block that is contaminated by random noise, *Srand*(*n*, *i*, *j*) is random noise with the value of +1 or −1 randomly.

(40) $$C^{\prime }(n,i,j)=C(n,i,j)+Srand(n,i,j)\cdot JND$$

Table 4 lists the comparison of the Kim’s CM-JND and the proposed *J_GDE-S_* models in terms of PSNR and DMOS values. As shown in Table 4, the average PSNR values of the Kim’s CM-JND and the proposed JND model are 52.77 dB and 49.82 dB respectively, and their corresponding DMOS values are both −0.1. That is, the proposed JND model produces 2.95 dB lower average PSNR at the same visual quality level compared with the Kim’s CM-JND model. The DMOS value of −0.1 indicates that most of subjects rarely distinguish the distorted images compared to the original ones. The results show that the proposed *J_GDE-S_* model is more appropriate to image with 10-bit depth than Kim’ JND model.

### 4.2. Objective and Subjective Performance Evaluation for the Proposed PVC Scheme

To verify the effectiveness of the proposed HEVC-compliant PVC scheme, the proposed *J_GDE-S_* model is implemented into HM 16.9, and it is compared with the original HM 16.9 and Kim’s PVC scheme [21]. Because Kim’s PVC is proposed in harmonization with HM 11.0 and also used in the RDO-based encoding process, the Kim’s PVC is re-implemented into HM 16.9 for fair comparison in this paper. The test sequence used for the experiments include eight different scenes of 4k UHD video sequences in the 4:2:0 color format. Because 10-bit depth is used as the mainstream for 4K UHD videos, the LD and RA configuration based on 10-bit depth are used for all experiments with a set of fixed quantization parameters, such as QP = 22, 27, 32, and 37.

We compare the original HM 16.9, Kim’s PVC and proposed PVC in terms of bitrate reduction and encoding time to verify the objective RD performance and encoder complexity. Then we also use DMOS values to verify subjective quality assessment. The bitrate reduction is represented as (41), between the original HM16.9 and the proposed (Kim’s and our) PVC scheme.

(41) $$\Delta R=\frac{R_{PVC}-R_{ori}}{R_{ori}}\times 100\%$$

and encoding time is represented as:

(42) $$\Delta T=\frac{T_{PVC}-T_{ori}}{T_{ori}}\times 100\%$$

where *R_ori_* and *T_ori_* are the bitrates and encoded time produced by the original HM 16.9. where *R_PVC_* and *T_PVC_* are the bitrates and encoded time produced by proposed or Kim’s PVC scheme. The proposed PVC scheme are also compared via subjective quality assessment experiments. For this, the DSCQS method is employed for subjective evaluation and the experimental set as Section 4.1. For movie video sequence, A and B are respectively presented to last 10 s and 3 s dummy videos are inserted between them as Figure 16 shows, where one test sequence is a reconstructed sequence encoded by the original HM 16.9, and the other is a reconstructed sequence encoded by proposed PVC or Kim’s PVC. The presentation order of the two test sequences are randomly selected for each presentation. The subjective voting scores are set the same as Section 4.1.

Table 5, Table 6 and Table 7 show the objective and subjective test results for the proposed PVC scheme, the Kim’s PVC scheme and the original HM 16.9 under LD and RA Main10 profiles, respectively. For performance in terms of bitrate reduction, the proposed PVC scheme outperforms Kim’s PVC scheme for all the test sequences and all the four QP values, yielding the average ∆*R* = 32.98%, the maximum ∆*R* = 80.89% under the LD profile and the average ∆*R* = 28.61%, the maximum ∆*R* = 66.04% under the RA profile for the ‘DaylightRoad2’ sequence at QP = 22. The maxim bitrate reduction obtained from the ‘DaylightRoad2’ sequence is due to the fact that it contains richer scenes and a lot of complex texture regions throughout all the frames. Therefore, it contains more noise which is insensitive to visual perception so that the proposed PVC scheme effectively suppresses the transform coefficients in those regions.

Table 5 and Table 6 show that the ∆*R* values are usually decreased as QP values increase for both PVC schemes. This is because the distortions introduced by quantization errors are high enough with QP values increasing, in order to maintain high objective quality, thus resulting in almost no room for coefficient suppression and making the coefficient suppression relatively less contribute to the total bitrate reduction. Compared to the Kim’s PVC scheme, the proposed PVC scheme achieves remarkably higher bitrate reductions with higher objective and subjective quality than Kim’s for all test sequences at different QPs. In order to reach a large extent of suppression for DCT coefficients, the JND threshold values are scaled to a large threshold values in Kim’s PVC. Therefore, the reconstruction distortions of TU residual block are increased to cause the lower objective and subjective quality than proposed PVC scheme. Given that the DCCF is utilized in the proposed PVC scheme to control the extern of distortions, the proposed PVC scheme more effectively suppresses the transform coefficients than the Kim’s JND suppression. In particular, this is because the objective and subjective video quality encoded by proposed PVC scheme is higher than Kim’s PVC scheme.

It is noticed that the average bitrate reduction ratios for the test PVC schemes under the RA profile are slightly smaller than those obtained under the LD profile. This is because the encoding under the RA profile usually operates with smaller prediction errors than that under the LD profile. That is, there exist smaller chances for both the proposed *J_GDE-S_* suppression and Kim’s suppression on the transform coefficients of residues under the RA profile.

As shown in Table 5 and Table 6, the proposed PVC scheme increases the total encoding time only with average 12.94% and 22.45% under LD Main10 and RA Main10 profiles compared to the original HM 16.9, respectively. Because the temporal masking is not considered and the proposed simple distortion compensation factor is utilized in our PVC scheme, the proposed PVC scheme is lower in complexity than Kim’s.

**Figure 17:**
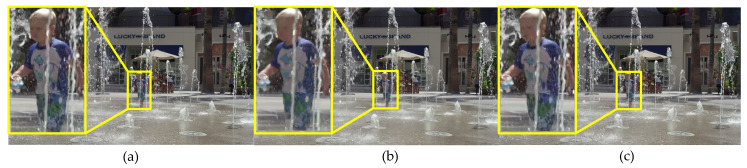
The 12th encoded frame of ToddlerFountain2 video sequence with QP = 32 under the LD mian10 configuration. (**a**) The encoded image with original HM 16.9, (**b**) the encoded image with Kim’ PVC, (**c**) the encoded image with proposed PVC.

**Figure 18:**
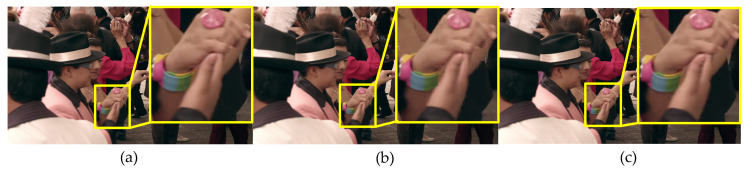
The 47th encoded frame of Tango2 video sequence with QP = 32 under the RA mian10 configuration. (**a**) The encoded image with original HM 16.9, (**b**) the encoded image with Kim’s PVC, (**c**) the encoded image with the proposed PVC.

Table 7 shows that the PSNR is a little degradation than the original PSNR for the proposed PVC scheme, and Kim’s PVC scheme. However, the PSNR of the proposed PVC scheme is higher than Kim’s. Usually the objective quality is not very well correlated to perceived visual quality, thus subjective quality is also an important measurement to assessment PVC scheme. For the subjective test results shown in Table 7, Kim’s and the proposed PVC scheme have statistically almost zero average DMOS values, yielding rarely visible distortion. The subjective quality of the proposed PVC scheme is higher than Kim’s PVC scheme. For example, the character details and edges are clearer compare with Kim’s PVC scheme in the yellow boxes of Figure 17 and Figure 18, due to the effect of different QPs on distortion considered in the proposed PVC scheme.

From the objective and subjective test results, the proposed PVC scheme achieves significant bitrate reduction at the same perceptual quality with little increment in encoder complexity compared to the original HM 16.9, and outperforms the Kim’s PVC scheme about two times the average bitrate reduction ratio. The superiority of the proposed PVC scheme comes from the fact that the proposed JND model is more appropriate to UHD videos with 10-bit depth and the designed DCCF which minimize the bitrate for different QPs under high PSNR. Thus, the proposed PVC scheme can reduce more bitrates than Kim’s PVC scheme under the higher PSNR.

## 5. Conclusions

A perceptual friendly JND-based HEVC-compliant PVC scheme is proposed in this paper. To better reflect the UHD image/video perception characteristics of HVS, a simple *J_GDE-S_* model is proposed by appropriately subjective experiments. For example, a LM effect for UHD image/video with 10-bit depth is modeled, and it is suppressed by GDE filter which control the extent of imperceptible noise for image. In addition, a DCT-based saliency factor is added into proposed JND model according to the FOV of UHD image/video. A simple transform coefficient suppression method with JND model is proposed in harmonization with the transform and quantization process of HM16.9 in a HEVC-complaint manner. Perceptual distortion is also appropriately compensated in the RDO process by incorporating the proposed DCF. In order to efficiently control the extent of the distortion compensation based on different QPs, the DCCF is also incorporated to RDO process. In objective and subjective experiments, the proposed HEVC-compliant PVC scheme yielded a remarkable bitrate reduction of 32.98% average for LD configuration and 28.61% average for RA configuration with a negligible subjective quality loss. It only causes an average of 12.94% and 22.45% encoding time increase under LD and RA configuration compared to the original HM 16.9, respectively. However, the proposed PVC scheme can reduce more bitrates with higher objective and subjective quality assessment and is computationally faster than the Kim’s PVC. There are still some shortages and improvements in this paper, and we conclude as follow: (1) The proposed JND model is not considered in the chroma domain. (2) We only considered that the encoding OPs lead to the different extent of perceptual distortion in the RDO process. The other encoding characters are explored in our future work.

## Figures and Tables

**Table 1:** Description of the symbols in Figure 1.

Symbol	Description
*H_CSF_*(*w_i,j_*, *φ_i,j_*)	Basic contrast sensitivity function (CSF) model
*MF_LM_*(*w_i,j_*, *μ_p_*)	Luminance masking effects
*J_LM_*	The proposed luminance masking effects
*H_GDE_*	The proposed Gauss differential entropy (GDE) filter
*S*(*w_i,j_*)	Modulation factor for saliency
*J_GDE-S_*	The proposed JND model
*w_i,j_*, *φ_i,j_*, *μ_p_*	Cycles per degree, Directional angle, Average pixel intensity of *N* × *N* TU block

**Table 2:** Experimental setup.

Display Information	Display	CHANGHONG (55Q3R)
	Type and Size	LED 55’’
	Resolution	3840 × 2160
Dynamic contrast ratio		7000:1
Number of subjects		15 subjects (male: 10, female: 5, average age: 25, min: 22, max age: 33)
Viewing distance		1.2 m
Ambient illuminance		200 lux
DCT transform option		Floating point, 8 × 8 block size
Test image size (**P2**)		256 × 256 pixels (covering a parafovea region)
Test patch size (**P1**)		32 × 32 pixels (resided in a fovea region)

**Table 3:** Statistics of image segmentation region.

One Frame of Ultra-High Definition (UHD) Video Sequence	Texture > *σ_th_*	Smooth ≤ *σ_th_*
Bosphorus (1920 × 1080-8bit)	10.9%	89.1%
Bosphorus (3840 × 2160-8bit)	3.9%	96.1%
Bosphorus (3840 × 2160-10bit)	96.0%	4.0%
ShakeNDry (1920 × 1080-8bit)	36.8%	63.2%
ShakeNDry (3840 × 2160-8bit)	11.9%	88.1%
ShakeNDry (3840 × 2160-10bit)	95.6%	4.4%
HoneyBee (1920 × 1080-8bit)	12.6%	87.4%
HoneyBee (3840 × 2160-8bit)	3.4%	96.6%
HoneyBee (3840 × 2160-10bit)	99.9%	0.1%

**Table 4:** Subjective experiments for 10-bit 4K UHD image.

Test Image (3840 × 2160)	10-Bit Depth			
	*Kim*		*Pro.*	
	*PSNR* (dB)	*Differential Mean Opinion Score (DMOS)*	*PSNR* (dB)	*DMOS*
**Beauty**	50.42	−0.1	48.25	−0.1
**Bosphorus**	53.56	−0.1	50.82	−0.0
**HoneyBee**	53.28	−0.0	49.05	−0.0
**Jockey**	53.03	−0.1	49.40	−0.1
**ReadySetGo**	52.75	−0.0	49.95	−0.1
**ShakeNDry**	52.78	−0.0	48.71	−0.0
**YachtRide**	53.62	−0.1	50.66	−0.1
**TrafficFlow**	52.74	−0.1	49.48	−0.1
**Campfire**	52.74	−0.0	52.08	−0.0
**Avg.**	52.77	−0.1	49.82	−0.1

**Table 5:** Bit rate and encoding complexity comparison of LD configuration.

Sequence	QP	*Bitrates* (kpbs)			∆*R* (%)		∆*T* (%)	
		*HM*	*Kim*	*Pro.*	*Kim*	*Pro.*	*Kim*	*Pro.*
**Tango2**	22	37,173	25,339	13,945	−31.83	−62.49	9.92	4.68
	27	11,282	9327	7441	−17.33	−34.05	17.61	8.70
	32	5715	5485	5150	−4.02	−9.89	24.93	22.54
	37	3205	3150	3139	−1.72	−2.06	24.94	23.95
**Drums100**	22	152,613	55,726	37,854	−63.49	−75.20	5.44	−12.7
	27	37,861	26,516	17,501	−29.96	−53.78	15.33	8.01
	32	15,041	13,747	12,419	−8.60	−17.43	24.39	20.10
	37	7441	7225	6850	−2.90	−7.94	25.62	22.19
**Campfire**	22	100,798	51,279	34,100	−49.13	−66.17	10.68	−6.70
	27	29,612	24,236	15,683	−18.15	−47.04	15.65	5.47
	32	13,141	12,453	11,208	−5.24	−14.71	29.09	18.88
	37	7359	7152	6585	−2.81	−10.52	30.24	22.13
**Toddler-Fountain2**	22	272,351	184,980	175,151	−32.08	−35.69	13.12	8.91
	27	133,188	79,831	78,904	−40.06	−40.76	16.49	10.88
	32	60,899	38,526	51,470	−36.74	−15.48	19.65	24.38
	37	26,596	18,690	24,087	−29.73	−9.43	22.75	27.09
**CatRobot**	22	44,763	25,649	14,713	−42.70	−67.13	7.62	−1.80
	27	12,316	9980	7414	−18.97	−39.80	10.76	10.24
	32	6082	5692	5097	−6.41	−16.20	25.85	21.83
	37	3306	3222	2930	−2.54	−11.37	26.01	23.85
**Traffic-Flow**	22	9848	6094	3448	−38.12	−64.99	9.63	1.49
	27	2855	2363	1616	−17.23	−43.40	15.54	15.54
	32	1254	1132	1085	−9.73	−13.48	25.35	21.52
	37	630	628	614	−0.32	−2.54	26.05	23.73
**Daylight-Road2**	22	83,299	27,497	15,922	−66.99	−80.89	7.84	−9.72
	27	14,549	9970	7173	−31.42	−50.70	15.26	7.48
	32	6608	5831	5127	−11.76	−22.41	26.19	21.88
	37	3364	3054	2881	−9.22	−14.36	27.49	24.45
**Roller-coaster2**	22	31,689	22,893	12,844	−27.76	−59.47	4.89	−2.20
	27	11,672	10,450	6333	−10.47	−45.74	13.10	5.32
	32	5200	5040	4457	−3.77	−14.29	23.31	19.22
	37	2688	2646	2528	−1.56	−5.95	24.89	22.84
**Avg.**	-	−	−	−	−21.02	−32.98	18.61	12.94

**Table 6:** Bit rate and encoding complexity comparison of RA configuration.

Sequence	QP	*Bitrates* (kpbs)			∆*R* (%)		∆*T* (%)	
		*HM*	*Kim*	*Pro.*	*Kim*	*Pro.*	*Kim*	*Pro.*
**Tango2**	22	35,848	22,115	16,659	−38.31	−53.53	17.74	8.34
	27	10,511	9224	7399	−12.24	−29.61	21.02	23.32
	32	5386	5067	4776	−5.92	−11.33	38.41	32.22
	37	3072	3040	2917	−1.04	−5.05	39.08	34.53
**Drums100**	22	125,145	70,489	45,109	−43.67	−63.95	8.65	−3.01
	27	36,429	26,449	21,078	−27.40	−42.14	20.26	18.51
	32	15,608	14,090	13,229	−9.73	−15.24	41.02	29.86
	37	8003	7870	7414	−1.61	−7.36	41.44	31.83
**Campfire**	22	79,100	50,370	34,004	−36.32	−57.01	11.22	1.93
	27	26,395	21,733	14,980	−17.66	−43.25	19.63	13.85
	32	11,414	10,365	9655	−9.19	−15.41	43.84	28.96
	37	6485	6223	5765	−4.04	−11.10	46.70	33.55
**Toddler-Fountain2**	22	236,874	171,444	151,512	−27.62	−36.03	25.56	10.90
	27	109,785	78,272	67,705	−28.70	−38.33	24.65	15.26
	32	49,085	36,675	41,910	−25.28	−14.62	25.54	30.67
	37	21,776	18,756	19,609	−13.87	−9.95	32.57	34.04
**CatRobot**	22	46,842	35,783	20,886	−23.61	−55.41	11.37	8.47
	27	13,245	11,526	9017	−12.98	−31.92	11.64	24.02
	32	6756	6242	5749	−7.61	−14.91	41.90	33.67
	37	3804	3764	3409	−1.05	−10.38	42.06	35.20
**Traffic-Flow**	22	14,151	11,277	5928	−20.31	−58.11	18.46	13.10
	27	3965	3339	2676	−15.79	−32.51	31.58	21.22
	32	1850	1754	1627	−5.19	−12.05	44.47	34.81
	37	971	942	923	−2.97	−4.94	44.17	36.76
**Daylight-Road2**	22	80,460	43,424	27,325	−46.03	−66.04	11.87	−2.45
	27	15,401	12,109	9201	−21.38	−40.26	28.38	18.03
	32	7242	6142	5873	−15.19	−18.90	37.68	28.90
	37	3821	3529	3365	−7.64	−11.93	38.57	32.01
**Roller-coaster2**	22	28,274	20,336	14,506	−28.08	−48.69	16.33	9.80
	27	11,185	9031	6887	−19.26	−38.43	17.73	19.30
	32	5319	5011	4544	−5.79	−14.57	38.31	30.30
	37	2891	2838	2816	−1.83	−2.59	40.75	30.66
**Avg.**	-	-	-	-	−16.79	−28.61	29.14	22.45

**Table 7:** Objective and subjective quality comparison of LD and RA configurations.

Configuration		*LD*					*RA*				
Sequence	QP	*PSNR* (dB)			*DMOS*		*PSNR* (dB)			*DMOS*	
		HM	*Kim*	*Pro.*	*Kim*	*Pro.*	HM	*Kim*	*Pro.*	*Kim*	*Pro.*
**Tango2**	22	40.46	38.93	38.91	−0.1	−0.1	40.31	38.80	39.26	−0.1	−0.1
	27	39.59	38.01	37.85	−0.6	−0.2	39.57	37.94	38.40	−0.6	−0.3
	32	38.46	36.63	37.56	−0.8	−0.1	38.56	36.64	37.85	−0.8	−0.1
	37	36.90	35.05	36.14	−0.8	−0.3	37.15	35.14	36.55	−0.8	−0.5
**Drums100**	22	42.31	37.08	38.05	0.0	0.0	41.47	37.21	38.63	0.0	0.0
	27	39.30	35.57	36.31	−0.2	−0.1	39.10	35.86	37.13	−0.2	−0.1
	32	36.97	34.01	35.91	−0.2	−0.2	37.08	34.24	36.23	−0.2	−0.1
	37	34.62	32.33	33.88	−0.4	−0.2	34.94	32.61	34.29	−0.4	−0.2
**Campfire**	22	40.28	36.00	36.46	−0.2	−0.2	39.32	35.76	36.50	−0.4	−0.3
	27	37.38	34.67	35.20	−0.3	−0.3	37.07	34.39	35.11	−0.3	−0.5
	32	35.67	33.24	34.86	−0.4	−0.4	35.43	32.94	34.59	−0.5	−0.5
	37	33.88	31.90	33.29	−0.4	−0.3	33.65	31.67	33.02	−0.5	−0.3
**Toddler-Fountain2**	22	40.78	32.13	36.29	0.0	0.0	39.78	31.68	35.37	0.0	0.0
	27	37.06	29.89	32.90	−0.2	−0.1	35.88	29.62	32.12	−0.2	−0.1
	32	33.52	28.29	32.41	−0.3	−0.2	32.53	29.63	31.52	−0.3	−0.2
	37	30.59	26.99	30.01	−0.5	−0.3	29.90	26.73	29.34	−0.5	−0.3
**CatRobot**	22	40.28	38.21	38.28	0.0	0.0	40.23	38.14	39.03	0.0	0.0
	27	38.96	37.11	36.95	−0.1	0.0	39.17	37.18	37.93	−0.3	0.0
	32	37.34	35.65	36.42	−0.3	0.2	37.75	35.82	37.02	−0.4	0.2
	37	35.31	33.84	34.56	−0.5	−0.1	35.90	34.06	35.26	−0.7	−0.1
**Traffic-Flow**	22	39.84	38.70	38.48	−0.1	−0.4	39.89	38.49	38.90	−0.1	−0.5
	27	38.62	37.80	37.46	−0.2	−0.4	38.82	37.66	37.97	−0.3	−0.5
	32	37.27	36.58	36.66	−0.1	−0.3	37.55	36.34	37.05	−0.6	−0.3
	37	35.63	35.01	35.12	0.0	−0.2	36.02	34.80	35.61	−0.7	−0.2
**Daylight-Road2**	22	38.04	36.03	36.13	0.0	0.0	37.91	36.02	36.63	0.0	0.0
	27	36.87	35.12	35.11	−0.1	0.0	36.95	35.30	35.84	−0.2	0.0
	32	35.65	33.98	34.75	−0.4	−0.1	35.89	34.26	35.23	−0.5	−0.1
	37	34.03	32.59	33.28	−0.5	−0.2	34.41	32.85	33.86	−0.7	−0.2
**Roller-coaster2**	22	45.17	41.14	41.18	−0.2	−0.1	44.76	41.22	42.22	−0.2	−0.1
	27	42.81	39.34	39.09	−0.4	−0.3	42.70	39.57	40.13	−0.5	−0.3
	32	40.19	37.40	38.54	−0.5	−0.3	40.33	37.59	39.06	−0.5	−0.3
	37	37.61	35.35	36.06	−0.6	−0.4	37.92	35.45	36.98	−0.7	−0.4
**Avg.**	-	37.86	35.14	36.07	−0.3	−0.2	37.75	35.18	36.39	−0.4	−0.2
